# Infectious Agents and Inflammation: The Role of Microbiota in Autoimmune Arthritis

**DOI:** 10.3389/fmicb.2017.02696

**Published:** 2018-01-16

**Authors:** Andrea Picchianti-Diamanti, Maria M. Rosado, Raffaele D’Amelio

**Affiliations:** ^1^Department of Clinical and Molecular Medicine, Sant’Andrea University Hospital, Sapienza University of Rome, Rome, Italy; ^2^Independent Researcher, Rome, Italy

**Keywords:** microbiota, autoimmune arthritis, infections, immunosuppressive therapy, biologics

## Abstract

In higher vertebrates, mucosal sites at the border between the internal and external environments, directly interact with bacteria, viruses, and fungi. Through co-evolution, hosts developed mechanisms of tolerance or ignorance toward some infectious agents, because hosts established “gain of function” interactions with symbiotic bacteria. Indeed, some bacteria assist hosts in different functions, among which are digestion of complex carbohydrates, and absorption and supply of vitamins. There is no doubt that microbiota modulate innate and acquired immune responses starting at birth. However, variations in quality and quantity of bacterial species interfere with the equilibrium between inflammation and tolerance. In fact, correlations between gut bacteria composition and the severity of inflammation were first described for inflammatory bowel diseases and later extended to other pathologies. The genetic background, environmental factors (e.g., stress or smoking), and diet can induce strong changes in the resident bacteria which can expose the intestinal epithelium to a variety of different metabolites, many of which have unknown functions and consequences. In addition, alterations in gut permeability may allow pathogens entry, thereby triggering infection and/or chronic inflammation. In this context, a local event occurring at a mucosal site may be the triggering cause of an autoimmune reaction that eventually involves distant sites or organs. Recently, several studies attributed a pathogenic role to altered oral microbiota in rheumatoid arthritis (RA) and to gut dysbiosis in spondyloarthritis (SpA). There is also growing evidence that different drugs, such as antibiotics and immunosuppressants, can influence and be influenced by the diversity and composition of microbiota in RA and SpA patients. Hence, in complex disorders such RA and SpA, not only the genetic background, gender, and immunologic context of the individual are relevant, but also the history of infections and the structure of the microbial community at mucosal sites should be considered. Here the role of the microbiota and infections in the initiation and progression of chronic arthritis is discussed, as well as how these factors can influence a patient’s response to synthetic and biologic immunosuppressive therapy.

## Microbiota and the Immune System

Host–microbe interactions are the result of a co-evolution process, in which both partners have set borders and have developed mechanisms of tolerance or ignorance. In this context, hosts were able to establish a continuous connection with symbiotic bacteria, representing for the host a “gain of function.” Bacteria provide help for different functions, including the digestion of complex carbohydrates as well as the absorption and supply of vitamins (LeBlanc et al., 2013). Worth mentioning is that, in the complex network of interactions of the microbiome, the environment should be taken into account since the outbreak of an infection and/or an inflammatory disease relies on the complex relationships between the microorganisms, the host, and the environment (such as dietary components, pollutants and even drugs) (Kim et al., 2016).

Microbiota are composed of archaea, bacteria, fungi, and viruses forming a complex ecosystem of which bacteria are the most well-characterized member. Any microbial imbalance resulting in a shift (i.e., loss or overgrowth of a species) and/or reduction in microbial diversity is defined as dysbiosis.

The mucosa has a dual function in our body. It works as a physical barrier, promoting pathogen exclusion, and is an absorption site that allows entry of food-derived metabolites and gas exchange. The mucosal immune system is not fully developed when we are born because many of the lymphoid structures, especially in the gut, are formed only upon stimulation through exposure to microorganisms. In fact, mice bred and maintained in germ-free conditions have small Payer’s patches and lack isolated lymphoid follicles (ILF) in their bowel. Furthermore, serum levels of IgM natural antibodies and IgA secreting cells in the spleen and intestine are reduced in germ free mice (Rhee et al., 2004). This phenotype is rescued by gut colonization once the animals are transferred from isolators to conventional housing and food. At birth, colonization plays a crucial role in inducing and shaping the development of the secondary lymphoid organs as well as setting-up thresholds of reactivity for both innate and acquired immune responses. Thus, the neonate microbiota intervenes with the fine balance amongst infection, inflammation, and tolerance that may “imprint” the immune system for life.

Recently, Vatanen et al. (2016) followed-up the gut colonization of children from birth to 3 years of age. Children from Finland, Estonia and Russia were selected who shared a human leukocyte antigen (HLA) distribution typical of individuals at risk for autoimmunity (Vatanen et al., 2016). The study showed that, depending on the lipopolysaccharide (LPS) subtype produced by different *Bacteroides* species, Toll-like receptor-4 (TLR4) could be either activated or inhibited. This had an impact on tolerance to endotoxin, and, consequently, strongly conditioned the genetic predisposition of the infants to type I diabetes (T1D). Thus, the presence of different LPS subtypes in the gut could partially explain the difference in prevalence of early onset autoimmune diseases in Finland and Estonia compared to Russia. In line with the hygiene hypothesis, this work adds another clue to the correlation between exposure or lack of exposure to specific microorganisms in early life and the increase of autoimmune diseases in western countries.

The microbiota is comprised of both pathogenic and non-pathogenic bacteria, however, this distinction is not absolute. A microorganism may behave as commensal or as pathogenic with regards to the dietary components, nutritional milieu, broad-spectrum antibiotic treatment, co-infection or genetic background of its host. Moreover, even though commensals are by definition not supposed to induce immune responses, and lamina propria-derived antigen-presenting cells traffic preferentially to mucosal-associated lymphoid tissue (MALT) in order to restrict systemic immune responses against the microbiota, it is possible to find antibodies specific to commensals in the serum of healthy people, as well as circulating T cells reactive against non-pathogenic bacteria (Macpherson et al., 1996; Ergin et al., 2011). Also, in specific genetic conditions, such as in individuals with defects in interleukin-17 (IL17) signaling pathway, commensal microorganisms become pathogenic through invasion, causing fungal disease (Puel et al., 2011). Another condition in which commensals may be harmful is in mucosal leakage. Changes of permeability at mucosal sites or altered immune functions strongly condition the persistence of antigenic stimulation, not only at the place of microbial entry but also in faraway locations, such as articular sites (Asquith et al., 2014). Moreover, in frail patients, the administration of *Saccharomyces cerevisiae* as a probiotic has been anecdotally reported to induce sepsis (Montineri et al., 2008; Eren et al., 2014).

It is worth mentioning here that the microbiota itself plays a role in regulating the growth of opportunistic enteric infections, a mechanism termed colonization resistance (Stecher and Hardt, 2011). Often antibiotic administration, by altering the equilibrium of the gut microbiota, leads to overgrowth of *Clostridium difficile*. This infection represents, nowadays, a major clinical problem because *C. difficile* is one of the bacterial species with higher antibiotic resistance. Microbiota transplantation from healthy donors, by rescuing microbial equilibrium, has been one of the most successful strategies to treat serious *C. difficile* infection (Hand, 2016).

Bacterial products, such as the previously mentioned LPS, are sensed by enterocytes through TLRs. Microbial stimulation of enterocytes induce the production of antimicrobial peptides (AMPs) and the fucosylation of small intestinal epithelial cells by Fucosyltransferase 2 (Fut2), both of which can limit specific infections (Pickard et al., 2014). TLRs regulate innate immune responses but are also crucial for epithelial cell homeostasis at mucosal sites. Altered immune function of TLRs, for example due to different polymorphisms, may change binding to bacterial products, secretion of cytokines or chemokines, which consequently interferes with acquired immune cell generation/migration and inflammation. Thus, it can contribute to the persistence of inflammatory diseases such as rheumatoid arthritis (RA) (Rogier et al., 2015).

In addition to bacteria, bacterial-derived products can also control, directly or indirectly, the function of both epithelial and inflammatory cells. For example, two important metabolites are the short-chain fatty acids (SCFAs) and the lipid mediator prostaglandin E2 (PGE2). Recognition of the SCFAs by innate immune cells is important to modulate inflammation in response not only to intestinal but even to articular damage (Maslowski et al., 2009). Commensals are also able to regulate the inflammatory activity of monocytes, by promoting the release of PGE2 that, in turn, down-modulates the activation of tissue-damaging neutrophils (Grainger et al., 2013).

The gut is the main site for the generation of the two most important T cell populations, the inducible regulatory T cells (iTregs) and CD4IL17-producing cells (Th17), both of which play critical roles in the development and persistence of autoimmune disorders. Colonization of the distal small intestine, by segmented filamentous bacteria (SFB), is fundamental to development of resident lamina propria dendritic cells (DCs) able to release IL6 and IL22 that trigger the loop of Th17-Treg cell in the newborn gut (Ivanov et al., 2009; Maslowski et al., 2009). Although many studies have addressed gut microbiota composition in humans, we still do not know which would be the “best/healthiest” microbial composition for the maintenance of the balance between regulatory (iTregs) and inflammatory (Th17) cells (Hand, 2016). Recent studies revealed that the homeostasis of the colonic Treg compartment relies more on the synergistic effects of different bacterial strains than on a single species of that strain. This observation strongly suggests the importance of the entire bacterial community in shaping the appropriate microenvironment that is able to sustain the production and maintenance of the anti-inflammatory environment (Atarashi et al., 2013).

## Oral/Gut Dysbiosis and Arthritis

Rheumatoid arthritis and Spondyloarthritis (SpA) are chronic inflammatory autoimmune diseases potentially leading to quality of life impairment and reduction in life expectancy. RA is characterized by the presence of autoantibodies [rheumatoid factor (RF) and anti-citrullinated protein antibodies (ACPAs)] and is characterized clinically by the emergence of erosive synovitis predominantly involving small joints, such as hands, wrists, and feet. The reported prevalence of the disease is 0.5–1% in the general population (Hayter and Cook, 2012).

Spondyloarthritis is a pleomorphic group of conditions that includes ankylosing spondylitis (AS), psoriatic arthritis (PsA), reactive arthritis (ReA), SpA associated to inflammatory bowel disease (IBD), acute anterior uveitis and axial non-radiographic SpA. The reported prevalence for AS is 2.4/1,000 (Dean et al., 2014). Arthritis is the most frequent extra-intestinal disease manifestation in patients with IBD (range 15–40%); conversely, up to 60% of patients with SpA present subclinical gut inflammation and 10% overt inflammation evolving to Crohn’s disease (Mielants et al., 1995; De Vos et al., 1996; Olivieri et al., 2014).

The evidence of a direct role for the gut microbiota in the development of autoimmune arthritis arises from experimental studies on germ-free mice in which a reduced severity and/or incidence of arthritis has been demonstrated (Carding et al., 2015).

However, in man, a direct correlation between gut dysfunction and systemic inflammation has been clearly established only in AS. Some studies by Ciccia et al. (2012) have identified several alterations of the ileum architecture in AS patients that may be the cause for chronic inflammation, both local and systemic. AS patients showed disruption of the basal membrane with compromised epithelial cell permeability, hyperplasia of goblet cells (with increased mucins production) (Ciccia et al., 2012) and activation of Paneth cells, producing high levels of adenosine monophosphate, such as α-defensin 5 (Ciccia et al., 2010) and proinflammatory cytokines, such as IL23 (Ciccia et al., 2009). These characteristics may be the cause or the consequence of dysbiosis, with changes in the diversity of the commensal bacteria (Costello et al., 2015). Increased intestinal permeability, probably determined by genetic factors such as HLA-B27 expression (Rosenbaum and Davey, 2011; Lin et al., 2014; Bowness, 2015), results in continuous antigenic stimulation with activation of effector T cells. These effector cells do not show a clearly defined Th17, Th1, and/or Th9 polarization, due to the over-expression of an IL23 response by Paneth cells (Ciccia et al., 2009). Worth noting is that IL23 can regulate the maturation of autoreactive Th17 cells but can also induce chronic inflammation by stimulating IL17, IL6, IL8, and tumour necrosis factor (TNF)-α production in neutrophils and macrophages. Furthermore, a link between IL23R polymorphisms and susceptibility to psoriasis, PsA and AS have been shown (Liu et al., 2008).

In patients with PsA, subclinical gut inflammation is characterized by the overexpression of IL9 and the generation of Th9, Th17, and Th22 responses. Th9 cells produced in the gut are found to be increased in the peripheral blood and they are able to migrate, enter synovial tissues and become established. In the meantime, gut-activated Paneth cells express both IL9 and IL9 receptors, creating a positive autocrine loop that perpetuates local inflammation (Ciccia et al., 2016).

Solid data suggest that alterations in the oral microbiota can influence both progression and disease outcome in RA patients (Diamanti et al., 2016; **Table 1**). It has been speculated that certain species of bacteria, mainly *Porphyromonas gingivalis* (PG) which may be present in the oral cavity, and are increased in patients affected by periodontitis, can induce loss of tolerance and lead to citrullination of several peptides and proteins (Wegner et al., 2010; Quirke et al., 2014; Ciccia et al., 2016).

**Table 1:** Most frequently reported dysbiosis in RA and SpA patients and the proposed pathogenic mechanisms.

Autoimmune arthritis	Dysbiosis		Pathogenic mechanisms
	Increased amount	Decreased amount	
Rheumatoid arthritis	*Porphyromonas gingivalis* (PG) (periodontitis induced by PG is a poor prognostic factor of response to anti-TNFα therapy) *Lactobacillus salivarius* (active RA) *Prevotella copri* spp. (RA) *Mycoplasma fermentans, Escherichia coli, Proteus mirabilis* (early RA) *Collinsella, Eggerthella, Faecalibacterium* (long-standing RA)	*Haemophilus* spp. (active RA) Bacteroidetes *phylum* members (RA) Common commensals such as: *Bifidobacteria and Bacteroides* (early RA)	PG peptidyl-arginine-deaminase enzyme can catalyze citrullination on different self-proteins (mainly α-enolase and fibrinogen) thus resulting in the immune evasion to these neo-epitopes and production of ACPAs.
Spondyloarthritis	*Lachnospiraceae, Ruminococcaceae, Rikenellaceae, Porphyromonadaceae, Bacteroidaceae* (SpA) *Bifidobacterium* genus (children with enthesitis) The *genus Dialister* positively correlated with SpA activity Sulphate-reducing bacteria (AS) Firmicutes (skin of PsA patients)	*Veillonellaceae* and *Prevotellaceae* (SpA)* Faecalibacterium prausnitzii* spp. and *Lachnospiraceae* (children with enthesitis) *Akkermansia, Ruminococcus* and *Pseudobutyrivibrio* (stool of PsA patients) Actinobacteria, *Propionibacteria* spp (skin of PsA patients)	Disruption of the gut basal membrane with compromised epithelial cell permeability, hyperplasia of goblet cells and activation of Paneth cells. Continuous antigenic stimulation with activation of effector T cells.

*RA, rheumatoid arthritis; SpA, spondyloarthritis; PsA, psoriatic arthritis; ACPAs, anti-citrullinated protein antibodies.*

Citrullination is a post-translational modification that converts arginine residues into citrulline leading to relevant changes in the protein structure, charge and function. *Porphyromonas gingivalis* peptidyl-arginine-deiminases enzyme (PPAD) can catalyze citrullination on different self-proteins (mainly α-enolase and fibrinogen) thus resulting in immune evasion by these neo-epitopes and the production of ACPAs (McInnes and Schett, 2011).

Indeed, it has been reported a direct correlation exists between the serum level of antibodies to PG and ACPAs in RA patients (Mercado et al., 2001; Dissick et al., 2010; Scher et al., 2012; Lange et al., 2016). This observation has been confirmed by the increased prevalence of PG-induced periodontitis in RA patients as compared to healthy individuals. Likewise, other studies have demonstrated that the presence of periodontitis is a poor prognostic factor in RA patients treated with anti-TNFα agents. The clearing of a periodontal infection can reduce the severity of active RA and lead to a better disease outcome during synthetic and biologic immunosuppressant treatment (Ortiz et al., 2009; Savioli et al., 2012; Kaur et al., 2014).

In line with the above described observations, a case of complete and long-lasting recovery in a patient with early RA after PD treatment has been recently reported, suggesting that, at the onset of disease, periodontitis treatment may avoid the development of a chronic and progressive arthritis (Salemi et al., 2014).

The influence of the intestinal microbiota on RA is still a matter of debate. A lower representation of common commensals such as *Bifidobacteria and Bacteroides* species (Vaahtovuo et al., 2008) and an increase in *Mycoplasma fermentans* (Johnson et al., 2000), *Escherichia coli* (Newkirk et al., 2010) and *Proteus mirabilis* (Senior et al., 1999) has been reported in early RA patients, whereas another study showed that stool samples from early RA patients had significantly more *Lactobacillus* communities than healthy subjects (Liu et al., 2013). In patients with long-standing RA a decreased gut microbial diversity seems to correlate with disease duration and autoantibody levels as well as expansion of rare genera, *Collinsella, Eggerthella*, and *Faecalibacterium* (Chen et al., 2016). Other authors have reported, in the fecal microbiota of RA patients, an increase of *Prevotella copri* spp. with concomitant reduction of Bacteroidetes phylum members (Scher et al., 2013). Recently Zhang et al. (2015) described a dysbiosis of both fecal and oral samples from RA patients using metagenomic shotgun sequencing and a metagenome-wide association study; these concordant abnormalities in mouth and gut microflora were restored after immunosuppressive treatment. In particular, *Haemophilus spp*. was strongly reduced, whereas *Lactobacillus salivarius* was increased especially in oral microbiota of patients with active RA disease (Zhang et al., 2015; **Table 1**).

The role of the gastrointestinal tract in the pathogenesis of SpA was proposed by Costello et al. (2015) who have evaluated biopsy specimens from 9 early SpA patients finding a higher abundance of five families of bacteria (*Lachnospiraceae, Ruminococcaceae, Rikenellaceae, Porphyromonadaceae*, and *Bacteroidaceae*), accompanied by a reduction of *Veillonellaceae* and *Prevotellaceae* with respect to healthy controls. Tito et al analyzed the gut microbiota (colon biopsy specimens) of 27 patients with SpA and 15 healthy controls and showed that the intestinal inflammation was associated with the mucosal microbiota composition. Of particular interest, they found that the genus *Dialister* positively correlated with the SpA activity (Tito et al., 2016). Stebbings et al. (2002) were the first group that reported differences in the fecal samples of AS patients compared to healthy controls by using molecular analysis. They did not find significant differences in the microbiota composition but did find a higher proportion of sulfate-reducing bacteria in AS patients (Stebbings et al., 2002). Fecal samples and blood specimens obtained from children with enthesitis showed less *Faecalibacterium prausnitzii* spp. and *Lachnospiraceae* family members and a significant increase in the *Bifidobacterium* genus (Stoll et al., 2014). Finally, other authors have reported a significant increase in Firmicutes associated with a reduction in members of Actinobacteria *phylum* and *Propionibacteria* spp in the skin samples of patients with psoriasis, compared to healthy controls (Fahlén et al., 2012). The gut microbiota observed in patients with PsA also showed limited diversity compared to healthy controls. In particular, it has been demonstrated that there is a decrease in the bacterial genus *Akkermansia, Ruminococcus*, and *Pseudobutyrivibrio* in fecal samples from PsA patients (Scher et al., 2015; **Table 1**).

There is a growing awareness that the microbiota diversity and composition can influence a patient’s response to synthetic and biologic immunosuppressive therapy. Immunosuppressive drugs, such as cyclophosphamide and methotrexate (MTX), can induce a diffuse depletion of the gut microbiota associated with a decrease in the commensal anaerobic species in favor of potential pathogens that can damage the gut barrier, altering epithelial cell permeability with consequent bacterial translocation (Viaud et al., 2013; Karin et al., 2014). Different authors have underlined the importance of an intact intestinal microbiota in the response to sulphasalazine (SSA), a synthetic drug frequently used in autoimmune arthritis. They found a decreased efficacy of SSA therapy in patients with ulcerative colitis previously treated with severe antibiotic regimens (Das et al., 1973; Peppercorn and Goldman, 1972). Conversely, the use of a multi-strain probiotic regimen does not seem to interfere with the metabolism of SSA in RA patients (Lee et al., 2010).

By now, data on the impact of microbiota in patients receiving immunosuppressive biologics are limited and only refer to IBD patients. One research group has found a reduction in the bacterial species Firmicutes *phylum* in patients affected by relapsing Crohn’s disease as compared to clinically stable patients with administration of anti-TNFα agents. This modification in gut microbiota was associated with a reduction in *F. prausnitzii* spp. predicting clinical relapse of the Crohn’s disease (Rajca et al., 2014). More recently, Busquets et al. (2015) have demonstrated that adalimumab (fully human anti-TNFα monoclonal antibody) could partially restore a healthy microbiota in a small group of patients affected by Crohn’s disease. Treatment resulted in the recovery of Firmicutes, Bacteroides and Actinobacteria diversity species whereas *E. coli* spp. was decreased (Busquets et al., 2015).

## Infections and Arthritis

In autoimmune arthritis, such as RA and SpA, the hypothesis of an infectious event as the cause for disease induction and relapse has been under consideration for a long time. Arleevskaya et al. (2014) have shown during a 10-year follow-up study that minor infections are more frequent and prolonged in RA patients and their relatives than in families without a history of autoimmunity. This study suggests a role for some predisposing defects of anti-bacterial defense mechanisms, more than for a specific microorganism. However, although by now no causative pathogen has been clearly identified nor a direct correlation with a specific infection has been established, it does not imply its absence. In fact, many pathogens have been indicated as possible triggers for RA disease but heterogeneity of the studies impairs forming any conclusions (Arleevskaya et al., 2016).

The most intriguing and supported idea for a role of infection in triggering RA, is that the microorganism can enter to and be located in the intestine, lungs or mouth; where it can interact with environmental factors such as smoke and drugs and, with a genetic predisposition, lead to mucosal inflammation. This, in turn, results in increased immune activation and spreading of inflammatory mediators, which locally perpetuate the disease. Exacerbated immune activation may also lead to aberrant migration of intestinal macrophages and lymphocytes from inflamed mucosa to the joints (Salmi et al., 1995).

The complex and interwoven link between microbes and joint inflammation is a frequent challenge for clinicians.

A severe increase in the serologic inflammatory parameters (i.e., erythrocyte sedimentation rate and C reactive protein), induced by a concomitant infection in a patient with autoimmune arthritis under immunosuppressive therapy is commonly encountered in the rheumatologic clinical practice. Indeed, the stimulation of systemic inflammation induced by the infectious pathogen can resemble a reactivation of the autoimmune arthritis, leading clinicians to increase the immunosuppressive therapy with a consequent worsening and dissemination of the infection.

The link between microbes and arthritis also has a broad spectrum of clinical expression. In fact, it can range from septic arthritis, in which the pathogen is directly responsible for joint inflammation and damage, to ReA. In the latter, infections by microorganisms, mainly Gram negative, such as *Chlamydia trachomatis, Yersinia, Salmonella, Shigella, and Campylobacter* (Espinoza and García-Valladares, 2013), find a genetically predisposed background (i.e., HLAB27) eventually resulting in chronic inflammation. However, the mechanism underlying the immune stimulation and consequent migration to distant synovial tissue is poorly understood (Keat et al., 1978; Granfors et al., 1990; Merilahti-Palo et al., 1991). It is worth mentioning that T cell clones specific for enteric bacteria have been found in synovial tissue of patients with ReA (Hermann et al., 1990; Probst et al., 1993).

It can be argued that the early recognition and eradication of the infective focus can lead to a resolution of the inflammatory stimulus and consequent interruption of the chronic immune loop. This can be the case of periodontal treatment in early RA, antibiotic therapy for beta-hemolytic *Streptococcus* in rheumatic fever and anti-hepatitis C virus (HCV) treatment for mixed cryoglobulinemia.

Recently, Campisi et al. (2016) using a complex and elegant set of experiments in mice, demonstrated that microbial infection at mucosal sites can elicit a break in tolerance by promoting the presentation of self-antigens derived from epithelial cell apoptotic bodies. Apoptosis of infected colonic cells leads to the generation of both anti-pathogen and autoreactive T cells, mainly Th17 cells, with production of auto-antibodies and inflammation (Campisi et al., 2016). These results have important implications for understanding how infections can stimulate the development of autoimmunity in an immunological context different from bystander activation of autoreactive T cells or epitope mimicry. Further studies are needed to establish similar correlations in humans.

The risk of infections in autoimmune patients is higher than in healthy individuals, due to endogenous (dysfunctional immune system) and external factors (i.e., comorbidities and immunosuppressive therapy). The risk has been reported to be approximately double for infections of bone and joints, skin, soft tissues and respiratory tract (Myllykangas-Luosujarvi et al., 1995). Moreover, it is known that infections can induce disease relapses, present a more severe clinical outcome and can be a frequent cause of death in immune-suppressed subjects (Gonzalez et al., 2007).

Immunosuppressive treatment is the main exogenous factor contributing to the increased risk of infections in autoimmune patients. TNFα blocking agents are the first biological immunosuppressive therapy approved for both RA and SpA. Currently five different drugs targeting TNFα are available: infliximab (the first antibody to be approved) is a chimeric monoclonal antibody (mAb) with approximately one third murine and two thirds human sequences; adalimumab, a fully human mAb produced by chinese hamster ovary (CHO) cells; golimumab a fully human mAb; certolizumab pegol, a poly-etyhlene-glycol (PEG)ylated humanized Fab’ fragment; and etanercept a fusion protein that consists of the p75 part of TNFR2 and a human IgG1 Fc domain (Salemi et al., 2015; Dieterich et al., 2016). Although these agents have shown a good safety and efficacy profile, up to 40% of the patients may present a primary or secondary drug failure or treatment-related adverse events (Day, 2002).

In the last decade, several research groups have studied in depth the molecular pathways involved in the development of autoimmune arthritis, allowing identification of new therapeutic targets. Among those, the anti-CD20 chimeric mAb (rituximab) (de la Torre et al., 2012); the humanized anti-human IL6 receptor mAb (tocilizumab) (Nishimoto et al., 2007), and the CTLA-4-Ig (abatacept), a fusion protein that works as an inhibitor of T cell activation (Moreland et al., 2006), are currently available for RA patients. More recently, mAb against the p40 subunit of interleukin 12/23 ustekinumab (Gottlieb et al., 2009) and the mAb against the IL17A, secukinumab have been introduced for the treatment of patients with SpA (Patel et al., 2013).

Different authors have evaluated the infection risk induced by corticosteroids (CS) as well as synthetic and biologic immunosuppressant drugs in SpA and RA patients. Studies generally showed that synthetic immunosuppressive therapy (mainly MTX), is relatively safe, whereas data on the possible increase in the number and/or severity of infections in patients taking TNFα blocking agents are contrasting. Doran et al. (2002) showed that the use of CS, older age, extra-articular manifestations of RA, leukopenia, and comorbidities, are strong predictors of infections, whereas the use of synthetic immunosuppressive agents was not associated with increased risk. These results were partially confirmed by Lacaille et al. (2008), who reported an increased risk of infections in RA patients taking CS, but not synthetic immunosuppressive therapy. Data on the infection risk for biologic drugs derives mainly from national registries among which are the British Biologics Register and the RABBIT German Registry. Data from these registries estimated a two-fold increased risk of serious infections which was directly related to the dose of CS, mainly when CS were associated with TNFα inhibitors (Dixon et al., 2006; Zink et al., 2014). Results obtained from meta-analysis studies have also indicated a significant increase in the occurrence of serious infections (40%), in patients receiving anti-TNFα agents, while the data for opportunistic infections were not conclusive (Bongartz et al., 2006; Minozzi et al., 2016).

We have recently described, in a retrospective observational cohort study, that the combination of anti-TNFα with CS appears to be the most “pro-infective” treatment, whereas synthetic immunosuppressant drugs, either alone or associated with CS, were relatively safe in both patients with RA or with SpA (Germano et al., 2014). In contrast with these real-life data, the placebo-controlled trials evaluating TNFα blockers in RA patients, did not find a higher rate of infections in treated patients with respect to the placebo group (Lipsky et al., 2000; Klareskog et al., 2004; Weinblatt et al., 2005).

## Conclusion

Patients with autoimmune arthritis are at high risk of infections, due to endogenous (dysfunctional immune system) and external factors (i.e., comorbidities, drugs).

Infections can induce disease relapses and be characterized by a severe clinical outcome in these patients, representing a frequent cause of death. Furthermore, the stimulation of systemic inflammation induced by the infectious pathogen, mimicking a reactivation of the autoimmune arthritis, frequently represents a confounding and dangerous factor for clinicians.

In the last few years the efforts of several researchers have focused on the gut and mouth microflora leading to the identification of specific abnormalities in both its composition and diversity. In susceptible individuals, the presence of PG could be a trigger factor for RA development by inducing the generation of citrullinated proteins via a PPAD enzyme on different self-proteins that results in the production of ACPAs. In SpA patients the increased intestinal permeability, probably induced by genetic factors (e.g., HLA-B27) could lead to a disruption of the basal membrane, hyperplasia of goblet cells and activation of Paneth cells producing high levels of AMPs and IL23. It would result in a continuous antigenic stimulation with activation of effector T cells.

Careful monitoring for infectious foci, especially in the mouth and intestine, should be recommended in order to allow their early recognition and eradication, thus resulting in removal of the inflammatory stimulus, inhibition of the chronic immune loop and improvement of the arthritis.

## Author Contributions

AP-D and MR drafted the manuscript and performed the revision of the literature. RD critically revised the manuscript giving important intellectual contribution.

## Conflict of Interest Statement

The authors declare that the research was conducted in the absence of any commercial or financial relationships that could be construed as a potential conflict of interest.

## References

[B1] Arleevskaya M. I. Gabdoulkhakova A. G. Filina Y. V. Miftakhova R. R. Bredberg A. Tsybulkin A. P. (2014). A transient peak of infections during onset of rheumatoid arthritis: a 10-year prospective cohort study. *BMJ Open* 4:e005254. 10.1136/bmjopen-2014-005254 25180052 PMC4156809

[B2] Arleevskaya M. I. Kravtsova O. A. Lemerle J. Renaudineau Y. Tsibulkin A. P. (2016). How rheumatoid arthritis can result from provocation of the immune system by microorganisms and viruses. *Front. Microbiol.* 7:1296 10.3389/fmicb.2016.01296 27582741 PMC4987382

[B3] Asquith M. Elewaut D. Lin P. Rosenbaum J. T. (2014). The role of the gut and microbes in the pathogenesis of spondyloarthritis. *Best Pract. Res. Clin. Rheumatol.* 28 687–702. 10.1016/j.berh.2014.10.018 25488778 PMC4293151

[B4] Atarashi K. Tanoue T. Oshima K. Suda W. Nagano Y. Nishikawa H. (2013). Treg induction by a rationally selected mixture of Clostridia strains from the human microbiota. *Nature* 500 232–236. 10.1038/nature12331 23842501

[B5] Bongartz T. Sutton A. J. Sweeting M. J. Buchan I. Matteson E. L. Montori V. (2006). Anti-TNF antibody therapy in rheumatoid arthritis and the risk of serious infections and malignancies: systematic review and meta-analysis of rare harmful effects in randomized controlled trials. *JAMA* 295 2275–2285. 10.1001/jama.295.19.2275 16705109

[B6] Bowness P. (2015). HLA-B27. *Ann. Rev. Immunol.* 33 29–48. 10.1146/annurev-immunol-032414-112110 25861975

[B7] Busquets D. Mas-de-Xaxars T. López-Siles M. Martínez-Medina M. Bahí A. (2015). Anti-tumour necrosis factor. treatment with adalimumab induces changes in the microbiota of Crohn’s disease. *J. Crohns Colitis* 9 899–906 10.1093/ecco-jcc/jjv119 26142465

[B8] Campisi L. Barbet G. Ding Y. Esplugues E. Flavell R. A. Blander J. M. (2016). Apoptosis in response to microbial infection induces autoreactive TH17 cells. *Nat. Immunol.* 17 1084–1092. 10.1038/ni.3512 27455420 PMC5079524

[B9] Carding A. Verbeke K. Vipond D. T. Corfe B. M. Owen L. J. (2015). Dysbiosis of the gut microbiota in disease. *Microb. Ecol. Health Dis.* 26:26191 10.3402/mehd.v26.26191 25651997 PMC4315779

[B10] Chen J. Wright K. Davis J. M. Jeraldo P. Marietta E. V. Murray J. (2016). An expansion of rare lineage intestinal microbes characterizes rheumatoid arthritis. *Genome Med.* 8:43. 10.1186/s13073-016-0299-7 27102666 PMC4840970

[B11] Ciccia F. Accardo-Palumbo A. Alessandro R. Rizzo A. Principe S. Peralta S. (2012). Interleukin-22 and interleukin-22-producing NKp44Ϸ NK cells in the subclinical gut inflammation of patients with ankylosing spondylitis. *Arthritis Rheum.* 64 1869–1878. 10.1002/art.34355 22213179

[B12] Ciccia F. Bombardieri M. Principato A. Giardina A. Tripodo C. Porcasi R. (2009). Overexpression of interleukin-23, but not interleukin-17, as an immunologic signature of subclinical intestinal inflammation in ankylosing spondylitis. *Arthritis Rheum.* 60 955–965. 10.1002/art.24389 19333939

[B13] Ciccia F. Bombardieri M. Rizzo A. Principato A. Giardina A. R. Raiata F. (2010). Over expression of paneth cell derived antimicrobial peptides in the gut of patients with ankylosing spondylitis and subclinical intestinal inflammation. *Rheumatology* 49 2076–2083. 10.1093/rheumatology/keq239 20688803

[B14] Ciccia F. Guggino G. Ferrante A. Raimondo S. Bignone R. Rodolico V. (2016). Interleukin-9 overexpression and Th9 polarization characterize the inflamed gut,the synovial tissue, and the peripheral blood of patients with psoriatic arthritis. *Arthritis Rheumatol.* 68 1922–1931. 10.1002/art.39649 26895441

[B15] Costello M. E. Ciccia F. Willner D. Warrington N. Robinson P. C. Gardiner B. (2015). Intestinal dysbiosis in ankylosing & spondylitis. *Arthritis Rheumatol.* 67 686–691. 10.1002/art.38967 25417597

[B16] Das K. M. Eastwood M. A. McManus J. P. Sircus W. (1973). The metabolism of salicylazosulphapyridine in ulcerative colitis. I. The relationship between metabolites and the response to treatment in inpatients. *Gut* 14 631–641 4147555 10.1136/gut.14.8.631 PMC1412758

[B17] Day R. (2002). Adverse reactions to TNF-alpha inhibitors in rheumatoid arthritis. *Lancet* 359 540–541. 10.1016/S0140-6736(02)07718-8 11867103

[B18] de la Torre I. Leandro M. J. Edwards J. C. Cambridge G. (2012). Baseline serum immunoglobulin levels in patients with rheumatoid arthritis: relationships with clinical parameters and with B-cell dynamics following rituximab. *Clin. Exp. Rheumatol.* 30 554–560. 22510323

[B19] De Vos M. Mielants H. Cuvelier C. Elewaut A. Veys E. (1996). Long term evolution of gut inflammation in patients with spondyloarthropathy. *Gastroenterology* 110 1696–1703. 10.1053/gast.1996.v110.pm8964393 8964393

[B20] Dean L. E. Jones G. T. MacDonald A. G. Downham C. Sturrock R. D. Macfarlane G. J. (2014). Global prevalence of ankylosing spondylitis. *Rheumatology* 53 650–657. 10.1093/rheumatology/ket387 24324212

[B21] Diamanti A. P. Manuela Rosado M. Laganà B. D’Amelio R. (2016). Microbiota and chronic inflammatory arthritis: an interwoven link. *J. Transl. Med.* 14:233 10.1186/s12967-016-0989-3 27492386 PMC4973033

[B22] Dieterich W. Neurath M. F. Atreya R. (2016). Molecular mechanism of action of anti-tumor necrosis factor antibodies in inflammatory bowel diseases. *World J. Gastroenterol.* 22 9300–9313 27895418 10.3748/wjg.v22.i42.9300 PMC5107694

[B23] Dissick A. Redman R. S. Jones M. Rangan B. V. Reimold A. Griffiths G. R. (2010). Association of periodontitis with rheumatoid arthritis: a pilot study. *J. Periodontol.* 81 223–230. 10.1902/jop.2009.090309 20151800

[B24] Dixon W. G. Watson K. Lunt M. (2006). Rates of serious infection, including site-specific and bacterial intracellular infection, in rheumatoid arthritis patients receiving anti-tumor necrosis factor therapy. Results from the British society for rheumatology biologics register. *Arthritis Rheum.* 54 2368–2376 16868999 10.1002/art.21978

[B25] Doran M. F. Crowson C. S. Pond G. R. O’Fallon W. M. Gabriel S. E. (2002). Predictors of infection in Rheumatoid arthritis. *Arthritis Rheum.* 49 2294–2300. 10.1002/art.10529 12355476

[B26] Eren Z. Gurol Y. Sonmezoglu M. Eren H. S. Celik G. Kantarci G. (2014). Saccharomyces cerevisiae fungemia in an elderly patient following probiotic treatment. *Mikrobiyol. Bul.* 48 351–355 10.5578/mb.6970 24819274

[B27] Ergin A. Syrbe U. Scheer R. (2011). Impaired peripheral Th1 CD4^+^ T cell response to *Escherichia coli* proteins in patients with Crohn’s disease and ankylosing spondylitis. *J. Clin. Immunol.* 31 998–1009. 10.1007/s10875-011-9575-x 21901394

[B28] Espinoza L. R. García-Valladares I. (2013). Of bugs and joints: the relationship between infection and joints. *Reumatol Clin.* 9 229–238 10.1016/j.reuma.2012.06.008 22944142

[B29] Fahlén A. Engstrand L. Baker B. S. Powles A. Fry L. (2012). Comparison of bacterial microbiota in skin biopsies from normal and psoriatic skin. *Arch. Dermatol. Res.* 304 15–22. 10.1007/s00403-011-1189-x 22065152

[B30] Germano V. Cattaruzza M. S. Osborn J. Tarantino A. Di Rosa R. Salemi S. (2014). Infection risk in rheumatoid arthritis and spondyloarthropathy patients under treatment with DMARDs, corticosteroids and TNF-α antagonists. *J. Transl. Med.* 12:77 10.1186/1479-5876-12-77 24655394 PMC3994399

[B31] Gonzalez A. Kremers M. H. Crowson C. S. Nicola P. J. Davis J. M. III. Therneau T. M. (2007). The widening mortality gap, between rheumatoid arthritis patients and the general population. *Arthritis Rheum.* 56 3583–3587. 10.1002/art.22979 17968923

[B32] Gottlieb A. Menter A. Mendelsohn A. Shen Y. K. Li S. Guzzo C. (2009). Ustekinumab, a human interleukin 12/23 monoclonal antibody, for psoriatic arthritis: randomised, double-blind, placebo-controlled, crossover trial. *Lancet* 373 633–640. 10.1016/S0140-6736(09)60140-9 19217154

[B33] Grainger J. R. Wohlfert E. A. Fuss I. J. Bouladoux N. Askenase M. H. Legrand F. (2013). Inflammatory monocytes regulate pathologic responses to commensals during acute gastrointestinal infection. *Nat. Med.* 19 713–721. 10.1038/nm.3189 23708291 PMC3755478

[B34] Granfors K. Jalkanen S. Lindberg A. A. Mäki-Ikola O. von Essen R. Lahesmaa-Rantala R. (1990). *Salmonella* lipopolysaccharide in synovial cells from patients with reactive arthritis. *Lancet* 335 685–688; 10.1016/0140-6736(90)90804-E 1690327

[B35] Hand T. W. (2016). The role of the microbiota in shaping infectious immunity. Trends Immunol. 37 647–658. 10.1016/j.it.2016.08.007 27616558 PMC5048567

[B36] Hayter S. M. Cook M. C. (2012). Updated assessment of the prevalence, spectrum and case definition of autoimmune disease. *Autoimmun. Rev.* 11 754–765. 10.1016/j.autrev.2012.02.001 22387972

[B37] Hermann E. Mayet W. J. Poralla T. Meyer zum Büschenfelde K. H. Fleischer B. (1990). *Salmonella*-reactive synovial fluid T-cell clones in a patient with post-infectious *Salmonella* arthritis. *Scand. J. Rheumatol.* 19 350–355 10.3109/03009749009096790 2218431

[B38] Ivanov I. I. Atarashi K. Manel N. Brodie E. L. Shima T. Karaoz U. (2009). Induction of intestinal Th17 cells by segmented filamentous bacteria. *Cell* 139 485–498. 10.1016/j.cell.2009.09.033 19836068 PMC2796826

[B39] Johnson S. Sidebottom D. Bruckner F. Collins D. (2000). Identification of *Mycoplasma fermentans* in synovial fluid samples from arthritis patients with inflammatory disease. *J. Clin. Microbiol.* 38 90–93. 10618069 10.1128/jcm.38.1.90-93.2000 PMC86027

[B40] Karin M. Jobin C. Balkwill F. (2014). Chemotherapy, immunity and microbiota–a new triumvirate? *Nat. Med*. 20 126–127. 10.1038/nm.3473 24504404 PMC4339017

[B41] Kaur S. Bright R. Proudman S. M. Bartold P. M. (2014). Does periodontal treatment influence clinical and biochemical measures for rheumatoid arthritis? A systematic review and meta-analysis. *Semin. Arthritis Rheum.* 44 113–122. 10.1016/j.semarthrit.2014.04.009 24880982

[B42] Keat A. C. Maini R. N. Nkwazi G. C. Pegrum G. D. Ridgway G. L. Scott J. T. (1978). Role of Chlamydia trachomatis and HLA-B27 in sexually acquired reactive arthritis. *Br. Med. J.* 1 605–607 10.1136/bmj.1.6113.605 630254 PMC1603413

[B43] Kim K. S. Hong S. W. Han D. Yi J. Jung J. Yang B. G. Lee J. Y. (2016). Dietary antigens limit mucosal immunity by inducing regulatory T cells in the small intestine. *Science* 351 858–863. 10.1126/science.aac5560 26822607

[B44] Klareskog L. van der Heijde D. De Jager J. P. Gough A. Kalden J. Malaise M. (2004). Therapeutic effect of the combination of etanercept and methotrexate compared with each treatment alone in patients with rheumatoid arthritis: double-blind randomised controlled trial. *Lancet* 363 675–681. 10.1016/S0140-6736(04)15640-7 15001324

[B45] Lacaille D. Guh D. P. Abrahamowicz M. Anis A. H. Esdaile J. M. (2008). Use of non biologic disease-modifying antirheumatic drugs and risk of infection in patients with rheumatoid arthritis. *Arthritis Rheum.* 59 1074–1081. 10.1002/art.23913 18668604

[B46] Lange L. Thiele G. M. McCracken C. Wang G. Ponder L. A. Angeles-Han S. T. (2016). Symptoms of periodontitis and antibody responses to *Porphyromonas gingivalis* in juvenile idiopathic arthritis. *Pediatr. Rheumatol. Online J.* 14:8. 10.1186/s12969-016-0068-6 26861944 PMC4748489

[B47] LeBlanc J. G. Milani C. de Giori G. S. Sesma F. van Sinderen D. Ventura M. (2013). Bacteria as vitamin suppliers to their host: a gut microbiota perspective. *Curr. Opin. Biotechnol.* 24 160–168. 10.1016/j.copbio.2012.08.005 22940212

[B48] Lee H. J. Waller R. D. Stebbings S. Highton J. Orlovich D. A. Schmierer D. (2010). The effects of an orally administered probiotic on sulfasalazine metabolism in individuals with rheumatoid arthritis: a preliminary study. *Int. J. Rheum. Dis.* 13 48–54 10.1111/j.1756-185X.2009.01449.x 20374384

[B49] Lin P. Bach M. Asquith M. Lee A. Y. Akileswaran L. Stauffer P. (2014). HLA-B27 and human beta2-microglobulin affect the gut microbiota of transgenic rats. *PLOS ONE* 9:e105684. 10.1371/journal.pone.0105684 25140823 PMC4139385

[B50] Lipsky P. E. van der Heijde D. M. St Clair E. W. Furst D. E. Breedveld F. C. Kalden J. R. (2000). Anti-tumor necrosis factor trial in rheumatoid arthritis with concomitant therapy study group infliximab and methotrexate in the treatment of rheumatoid arthritis. *N. Engl. J. Med.* 343:1594–1602. 11096166 10.1056/NEJM200011303432202

[B51] Liu X. Zou Q. Zeng B. Fang Y. Wei H. (2013). Analysis of fecal lactobacillus community structure in patients with early rheumatoid arthritis. *Curr. Microbiol.* 67 170–176. 10.1007/s00284-013-0338-1 23483307

[B52] Liu Y. Helms C. Liao W. Zaba L. C. Duan S. Gardner J. (2008). A genome-wide association study of psoriasis and psoriatic arthritis identifies new disease loci. *PLOS Genet.* 4:e1000041 10.1371/journal.pgen.1000041 18369459 PMC2274885

[B53] Macpherson A. Khoo U. Y. Forgacs I. Philpott-Howard J. Bjarnason I. (1996). Mucosal antibodies in inflammatory bowel disease are directed against intestinal bacteria. *Gut* 38 365–375. 10.1136/gut.38.3.365 8675088 PMC1383064

[B54] Maslowski K. M. Vieira A. T. Ng A. Kranich J. Sierro F. Yu D. (2009). Regulation of inflammatory responses by gut microbiota and chemoattractant receptor GPR43. *Nature* 461 1282–1286 10.1038/nature08530 19865172 PMC3256734

[B55] McInnes I. B. Schett G. (2011). The pathogenesis of rheumatoid arthritis. *N. Engl. J. Med.* 365 2205–2219. 10.1056/NEJMra1004965 22150039

[B56] Mercado F. B. Marshall R. I. Klestov A. C. Bartold P. M. (2001). Relationship between rheumatoid arthritis and periodontitis. *J. Periodontol.* 72 779–787. 10.1902/jop.2001.72.6.779 11453241

[B57] Merilahti-Palo R. Söderström K. O. Lahesmaa-Rantala R. Granfors K. Toivanen A. (1991). Bacterial antigens in synovial biopsy specimens in yersinia triggered reactive arthritis. *Ann. Rheum. Dis.* 50 87–90 10.1136/ard.50.2.87 1998396 PMC1004343

[B58] Mielants H. Veys E. M. Cuvelier C. De Vos M. Goemaere S. De Clercq L. (1995). The evolution of spondyloarthropathies in relation to gut histology. II. Histological aspects. *J. Rheumatol.* 22 2273–2278. 8835561

[B59] Minozzi S. Bonovas S. Lytras T. Pecoraro V. González-Lorenzo M. Bastiampillai A. J. (2016). Risk of infections using anti-TNF agents in rheumatoid arthritis, psoriatic arthritis, and ankylosing spondylitis: a systematic review and meta-analysis. *Expert Opin. Drug Saf.* 15 (Suppl.1) 11–34. 10.1080/14740338.2016.1240783 27924643

[B60] Montineri A. Iacobello C. Larocca L. La Rosa R. Nigro L. Fatuzzo F. (2008). Saccharomyces cerevisiae fungemia associated with multifocal pneumonia in a patient with alcohol-related hepatic cirrhosis. *Infez. Med.* 16 227–229. 19155689

[B61] Moreland L. Bate G. Kirkpatrick P. (2006). Abatacept. *Nat. Rev. Drug Discov.* 5 185–186. 10.1038/nrd1989 16557658

[B62] Myllykangas-Luosujarvi R. Aho K. Kautiainen H. Isomaki H. (1995). Shortening of life span and causes of excess mortality in a population-based series of subjects with rheumatoid arthritis. *Clin. Exp. Rheumatol.* 13 149–153. 7656460

[B63] Newkirk M. M. Zbar A. Baron M. Manges A. R. (2010). Distinct bacterial colonization patterns of Escherichia coli subtypes associate with rheumatoid factor status in early inflammatory arthritis. *Rheumatology (Oxford)* 49 1311–1316. 10.1093/rheumatology/keq088 20360042

[B64] Nishimoto N. Hashimoto J. Miyasaka N. Yamamoto K. Kawai S. Takeuchi T. (2007). Study of active controlled monotherapy used for rheumatoid arthritis, an IL-6 inhibitor (SAMURAI): evidence of clinical and radiographic benefit from an x-ray reader-blinded radnomised controlled trial of tocilizumab. *Ann. Rheum. Dis.* 66 1162–1167. 10.1136/ard.2006.068064 17485422 PMC1955155

[B65] Olivieri I. Cantini F. Castiglione F. Felice C. Gionchetti P. Orlando A. (2014). Italian Expert Panel on the management of patients with coexisting spondyloarthritis and inflammatory bowel disease. *Autoimmun. Rev.* 13 822–830. 10.1016/j.autrev.2014.04.003 24726868

[B66] Ortiz P. Bissada N. F. Palomo L. Han Y. W. Al-Zahrani M. S. Panneerselvam A. (2009). Periodontal therapy reduces the severity of active rheumatoid arthritis in patients treated with or without tumor necrosis factor inhibitors. *J. Periodontol.* 80 535–540. 10.1902/jop.2009.080447 19335072 PMC2884010

[B67] Patel D. D. Lee D. M. Kolbinger F. Antoni C. (2013). Effect of IL-17A blockade with secukinumab in autoimmune diseases. *Ann. Rheum. Dis.* 72(Suppl. 2) 116–123. 10.1136/annrheumdis-2012-202371 23253932

[B68] Peppercorn M. A. Goldman P. (1972). The role of intestinal bacteria in the metabolism of salicylazosulfapyridine. *J. Pharmacol. Exp. Ther.* 181 555–562. 4402374

[B69] Pickard J. M. Maurice C. F. Kinnebrew M. A. Abt M. C. Schenten D. Golovkina T. V. (2014). Rapid fucosylation of intestinal epithelium sustains host-commensal symbiosis in sickness. *Nature* 514:638–641 10.1038/nature13823 25274297 PMC4214913

[B70] Probst P. Hermann E. Meyer zum Büschenfelde K. H. Fleischer B. (1993). Multiclonal synovial T cell response to Yersinia enterocolitica in reactive arthritis: the Yersinia 61-kDa heat-shock protein is not the major target antigen. *J. Infect. Dis.* 167 385–391 7678427 10.1093/infdis/167.2.385

[B71] Puel A. Cypowyj S. Bustamante J. Wright J. F. Liu L. Lim H. K. (2011). Chronic mucocutaneous candidiasis in humans with inborn errors of interleukin-17 immunity. *Science* 332 65–68. 10.1126/science.1200439 21350122 PMC3070042

[B72] Quirke A. M. Lugli E. B. Wegner N. Hamilton B. C. Charles P. Chowdhury M. (2014). Heightened immune response to autocitrullinated *Porphyromonas gingivalis* peptidylarginine deiminase: a potential mechanism for breaching immunologic tolerance in rheumatoid arthritis. *Ann. Rheum. Dis.* 73 263–269. 10.1136/annrheumdis-2012-202726 23463691 PMC3888615

[B73] Rajca S. Grondin V. Louis E. Vernier-Massouille G. Grimaud J. C. Bouhnik Y. (2014). Alterations in the intestinal microbiome (dysbiosis) as a predictor of relapse after infliximab withdrawal in Crohn’s disease. *Inflamm. Bowel Dis.* 20 978–986. 10.1097/MIB.0000000000000036 24788220

[B74] Rhee K. J. Sethupathi P. Driks A. Lanning D. K. Knight K. L. (2004). Role of commensal bacteria in development of gut-associated lymphoid tissues and preimmune antibody repertoire. *J. Immunol.* 172 1118–1124. 10.4049/jimmunol.172.2.1118 14707086

[B75] Rogier R. Koenders M. I. Abdollahi-Roodsaz S. (2015). Toll-like receptor mediated modulation of T cell response by commensal intestinal microbiota as a trigger for autoimmune arthritis. *J. Immunol. Res.* 2015:527696. 10.1155/2015/527696 25802876 PMC4352938

[B76] Rosenbaum J. T. Davey M. P. (2011). Time for a gut check: evidence for the hypothesis that HLA-B27 predisposes to ankylosing spondylitis by altering the microbiome. *Arthritis Rheum.* 63 3195–3198. 10.1002/art.30558 21792826 PMC3204318

[B77] Salemi S. Biondo M. I. Fiorentino C. Argento G. Paolantonio M. Di Murro C. (2014). Could early rheumatoid arthritis resolve after periodontitis treatment only? Case report and review of the literature. *Medicine* 93:e195. 10.1097/MD.0000000000000195 25501069 PMC4602768

[B78] Salemi S. Markovic M. Martini G. D’Amelio R. (2015). The expanding role of therapeutic antibodies. *Int. Rev. Immunol.* 34 202–264 10.3109/08830185.2013.863304 24471447

[B79] Salmi M. Andrew D. P. Butcher E. C. Jalkanen S. (1995). Dual binding capacity of mucosal immunoblasts to mucosal and synovial endothelium in humans: dissection of the molecular mechanisms. *J. Exp. Med.* 181 137–149. 10.1084/jem.181.1.137 7528765 PMC2191840

[B80] Savioli C. Ribeiro A. C. Fabri G. M. Calich A. L. Carvalho J. Silva C. A. (2012). Persistent periodontal disease hampers anti-tumor necrosis factor treatment response in rheumatoid arthritis. *J. Clin. Rheumatol.* 18 180–184. 10.1097/RHU.0b013e31825828be 22647860

[B81] Scher J. U. Sczesnak A. Longman R. S. Segata N. Ubeda C. Bielski C. (2013). Expansion of intestinal prevotella copri correlates with enhanced susceptibility to arthritis. *Elife* 2:e01202. 10.7554/eLife.01202 24192039 PMC3816614

[B82] Scher J. U. Ubeda C. Artacho A. Attur M. Isaac S. Reddy S. M. (2015). Decreased bacterial diversity characterizes the altered gut microbiota in patients with psoriatic arthritis, resembling dysbiosis in inflammatory bowel disease. *Arthritis Rheumatol.* 67 128–139. 10.1002/art.38892 25319745 PMC4280348

[B83] Scher J. U. Ubeda C. Equinda M. Khanin R. Buischi Y. Viale A. (2012). Periodontal disease and the oral microbiota in new-onset rheumatoid arthritis. *Arthritis Rheum.* 64 3083–3094. 10.1002/art.34539 22576262 PMC3428472

[B84] Senior B. W. Anderson G. A. Morley K. D. Kerr M. A. (1999). Evidence that patients with rheumatoid arthritis have asymptomatic ’non-significant’ *Proteus mirabilis* bacteriuria more frequently than healthy controls. *J. Infect.* 38 99–106. 10.1016/S0163-4453(99)90076-2 10342649

[B85] Stebbings S. Munro K. Simon M. A. Tannock G. Highton J. Harmsen H. (2002). Comparison of the fecal microflora of patients with ankylosing spondylitis and controls using molecular methods of analysis. *Rheumatology* 41 1395–1401. 10.1093/rheumatology/41.12.1395 12468819

[B86] Stecher B. Hardt W. D. (2011). Mechanisms controlling pathogen colonization of the gut. *Curr. Opin. Microbiol.* 14 82–91. 10.1016/j.mib.2010.10.003 21036098

[B87] Stoll M. L. Kumar R. Morrow C. D. Lefkowitz E. J. Cui X. Genin A. (2014). Altered microbiota associated with abnormal humoral immune responses to commensal organisms in enthesitis-related arthritis. *Arthritis Res. Ther.* 16:486. 10.1186/s13075-014-0486-0 25434931 PMC4272554

[B88] Tito R. Y. Cypers H. Joossens M. Varkas G. Van Praet L. Glorieus E. (2016). *Dialister* as a microbial marker of disease activity in spondyloarthritis. *Arthritis Rheumatol.* 69 114–121. 10.1002/art.39802 27390077

[B89] Vaahtovuo J. Munukka E. Korkeamäki M. Luukkainen R. Toivanen P. (2008). Fecal microbiota in early rheumatoid arthritis. *J. Rheumatol.* 35 1500–1505. 18528968

[B90] Vatanen T. Kostic A. D. d’Hennezel E. Siljander H. Franzosa E. A. Yassour M. (2016). Variation in microbiome LPS immunogenicity contributes to autoimmunity in humans. *Cell* 165 842–853 10.1016/j.cell.2016.04.007 27133167 PMC4950857

[B91] Viaud S. Saccheri F. Mignot G. Yamazaki T. Daillère R. Hannani D. (2013). The intestinal microbiota modulates the anticancer immune effects of cyclophosphamide. *Science* 342 971–976. 10.1126/science.1240537 24264990 PMC4048947

[B92] Wegner N. Wait R. Sroka A. Eick S. Nguyen K. A. Lundberg K. (2010). Peptidylarginine deiminase from *Porphyromonas gingivalis* citrullinates human fibrinogen and α-enolase: implications for autoimmunity in rheumatoid arthritis. *Arthritis Rheum.* 62 2662–2672. 10.1002/art.27552 20506214 PMC2941529

[B93] Weinblatt M. E. Keystone E. C. Furst D. E. (2005). Long-term efficacy and safety of adalimumab plus methotrexate in patients with rheumatoid arthritis: ARMADA 4-year extended study. *Ann. Rheum. Dis.* 65 753–759. 10.1136/ard.2005.044404 16308341 PMC1798163

[B94] Zhang X. Zhang D. Jia H. Feng Q. Wang D. Liang D. (2015). The oral and gut microbiomes are perturbed in rheumatoid arthritis and partly normalized after treatment. *Nat. Med.* 21 895–905. 10.1038/nm.3914 26214836

[B95] Zink A. Manger B. Kaufmann J. Eisterhues C. Krause A. Listing J. (2014). Evaluation of the RABBIT risk score for serious infections. *Ann. Rheum. Dis.* 73 1673–1676. 10.1136/annrheumdis-2013-203341 23740236 PMC4145466

